# A young‐onset type 2 diabetic Chinese girl with familial renal glycosuria caused by a novel mutation in *SLC5A2* : A case report

**DOI:** 10.1111/1753-0407.13410

**Published:** 2023-05-16

**Authors:** Yuqing Qu, Limei Hao, Xianling Wang

**Affiliations:** ^1^ Department of Endocrinology Yantai Yuhuangding Hospital Yantai China; ^2^ Department of Endocrinology The First Medical Center of Chinese PLA General Hospital Beijing China; ^3^ Department of Endocrinology Dingzhou People's Hospital Dingzhou China

## 1 INTRODUCTION

The pathogenesis of youth‐onset type 2 diabetes is different from that of maturity‐onset diabetes of the young (MODY) and from type 1 diabetes, but there are many overlapped clinical manifestations among these three types of diabetes. Delays in differential diagnosis and incorrect treatment will definitely cause worse prognosis. If hyperglycemia cannot be well controlled in diabetic patients, hyperglycosuria and even ketonuria will be present.

Proximal tubules in the kidneys can reabsorb 180 g glucose every day in physiological conditions. If plasma glucose concentrations are in the normal range (< 200 mg/dL), all the filtered glucose will be reabsorbed in the proximal renal tubules. As a result, no urinary glucose is excreted.^1^

Familial renal glycosuria (FRG) is a rare genetic disorder of proximal tubular glucose transport, characterized by excessive urinary glucose excretion even if blood glucose level is in normal range. It has been confirmed that mutations in the solute carrier family 5 members 2 (*SLC5A2*) gene, which encodes sodium‐glucose cotransporter 2 (SGLT2), are responsible for this disease.^2^ The *SLC5A2* gene is a 7.7 kb gene containing 14 exons and encoding protein SGLT2, with 672 amino acids and an inferred secondary structure consisting of 14 transmembrane‐spanning domains and expressed in the S1 and S2 segments of the proximal convoluted tubule, which account for 90% of renal glucose reabsorption.^3^ FRG may be transmitted in an autosomal dominant pattern, although current studies demonstrated that its inheritance may be codominant with incomplete penetrance. FRG is a benign condition, which does not need any treatment, and the prognosis of patients with this disorder is usually favorable.

There is no report about youth‐onset type 2 diabetes associated with FRG. Herein we described the clinical characteristics of a Chinese girl with youth‐onset type 2 diabetes coexisting with FRG, in whom a novel mutation (NM_003041.4:c.1129 + 2 T > C) of the *SLC5A2* gene might explain FRG occurrence.

## 2 METHODS

### 2.1 Patient

This research was approved by the Ethics Committee of the First Medical Center of Chinese PLA General Hospital and performed according to the approved guidelines. Written informed consent was obtained from the proband and her parents. Clinical and biochemical information was collected from all the family members.

### 2.2 Genetic analysis

Genomic DNA of all participants was extracted from peripheral blood by QIAamp DNA extraction kit (Germany QIAGEN company). The genomic DNA sample of the proband was fragmented with the DNase and amplified by polymerase chain reaction. The target region of the whole exome was captured and purified by IDT xGen Exome Research panel and sequenced on a NovaSeq 6000 sequencer (Illumina, USA) to provide mean sequence coverage of more than 90×, with more than 98% of the target bases having at least 20× coverage. Variant calling was obtained with the use of GATK (v4.0.6.0), and then ANNOVAR (v20211019) was used for variant annotation. Variant was compared with the list of reported mutations from the Human Gene Mutation Database (http://www.hgmd.cf.ac.uk), Online Mendelian Inheritance in Man, refseq database, dbSNP database, ClinVar database. The pathogenicity of the variant was classified according to the interpretation guidelines of the American College of Medical Genetics and Genomics (ACMG). According to the result of high‐throughput sequencing, the mutation of the proband and her parents was verified by Sanger sequencing.

## 3 RESULTS

### 3.1 Clinical Characteristics

The proband was a 20‐year‐old Chinese girl who was referred to the diabetes department for repeated episodes of glycosuria and ketonuria caused by diabetes lasting 6 years, the onset of which at age 14 years was characterized by polyuria, polydipsia, and weight loss. After admission in our hospital, endocrine evaluations were thoroughly performed. Her body mass index was 20.9 kg/m^2^ and blood pressure was in the normal range. Serum hemoglobin A_1c_ was 10.6% (reference range [RR] 4.1%–6.0%). The results of the mixed meal tolerance test showed fasting plasma glucose (FPG) 187.92 mg/dL (RR 61.2–109.8 mg/dL), 2‐h postprandial glucose (2‐h PG) 222.12 mg/dL, fasting C‐peptide 20.9 ug/L (RR 9.9–39.64 ug/L) and 2‐h postprandial C‐peptide 31.8 ug/L respectively. The islet autoantibodies (glutamic acid decarboxylase antibodies, islet cell antibody, and insulin autoantibodies) were all negative. Twenty genes associated with neonatal diabetes mellitus and 14 genes associated with MODY were investigated through whole exome sequencing. However, no mutation was found. Finally, she was diagnosed with youth‐onset type 2 diabetes. Initially, the proband achieved better glycemic control with lifestyle management. But 1 year later, hyperglycemia episodes became more serious. Hyperglycemia could not be well controlled with oral hypoglycemic agents. When her blood glucose was better controlled and maintained around the normal range with insulin therapy, the laboratory examination still showed continuous ketonuria (+, +++) and glycosuria (++++). Moreover, pyuria and bacteriuria were also detected on urinanalysis, for diagnosis of urinary tract infection (UTI), successfully treated with oral levofloxacin. The therapeutic regimen for metabolic homeostasis was based on insulin as part 30 injection (bid, 57 IU/d) and metformin (1.0 g, bid).

### 3.2 Clinical characteristics of the parents

Her parents did not suffer from any renal disease, and serum creatinine and urine protein excretion were normal. The 75 g oral glucose tolerance tests (OGTT) were performed on both of them. The FPG and 2‐h PG of her father were 108 mg/dL (RR 68.4–111.6 mg/dL) and 152.28 mg/dL, which indicated impaired glucose tolerance. However, the glycosuria qualitative test showed negative, ++++ and +++ at 0, 1, 2 h in 75 g OGTT. Meanwhile, 24‐h urine glucose excretion was 0.33 g/1.73 m^2^/24 h, which was not consistent with the level of blood glucose. Conversely, the FPG and 2‐h PG of her mother were 132.3 mg/dL (RR 68.4–111.6 mg/dL) and 276.84 mg/dL. The 24‐h urine glucose excretion was 0.12 g/1.73 m^2^/24 h. Because her mother had type 2 diabetes, glycosuria was consistent with the level of blood glucose.

### 3.3 Genetic Analysis

**FIGURE 1:**
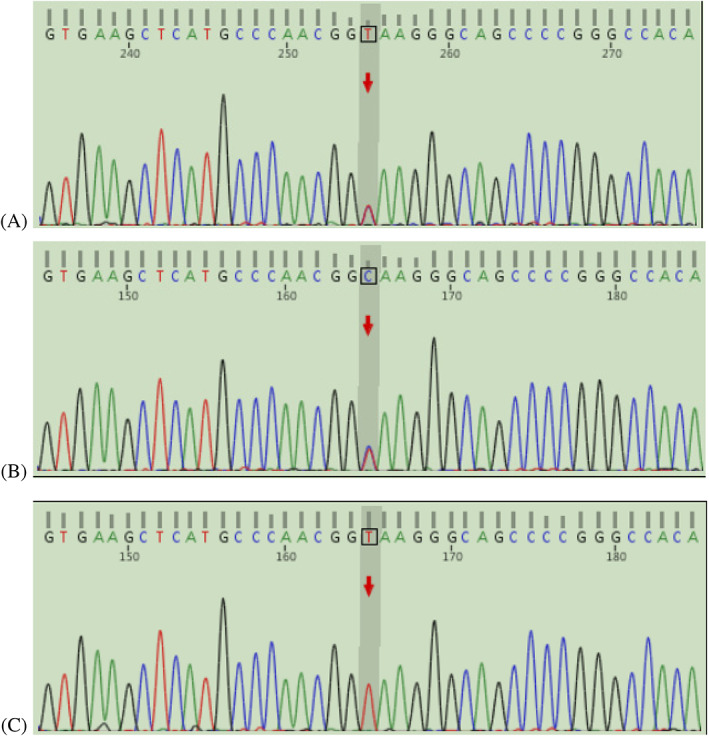
Mutation analysis of the solute carrier family 5 member 2 gene in family members affected with familial renal glycosuria. The positions of the mutations are indicated by the red arrows. (A) The proband harbored a compound heterozygous mutation, c.1129 + 2 T > C. (B) The father of the proband was detected to have an asymptomatic heterozygous mutation c.1129 + 2 T > C. (C) The mother of the proband carried the wild‐type sequence.

Fourteen coding exons in *SLC5A2* and their flanking intronic regions were analyzed (Figure 1). A T‐to‐C transition at complementary deoxyribonucleic acid (cDNA) (Genome Reference Consortium human genome build 37/human genome 19 16:31499813) of *SLC5A2* was identified in the proband. This variation was located in intron 9. The same mutation was detected in her father. Sanger sequencing did not reveal alterations of *SLC5A2* gene in her mother.

### 3.4 Follow‐up

The combination of metformin and large dose insulin (57 IU/d) still could not effectively maintain hyperglycemia under control. Even when her blood glucose level was near the normal range, ketonuria (fluctuating from + to +++) and glycosuria were still persistent. Moreover, UTI was recurrent, and the therapy could not be as effective as previously occurred.

## 4 DISCUSSION

The patient presented with polydipsia, polyuria, and weight loss at age 14 years. Endocrinological evaluations showed fasting and postprandial serum insulin and C‐peptide in the normal range, with negative islet autoantibodies, thus excluding type 1 diabetes diagnosis and presuming the MODY one.

MODY is a special type of diabetes mellitus, representing a small but very important fraction of diabetic patients (1%–5%),^4^, ^5^ and 1%–6% of cases in pediatric age.^6^ In this patient, no mutations in 14 genes associated with MODY have been detected, and its diagnosis was also excluded.

The clinical presentation of youth‐onset type 2 diabetes resembles that of MODY, but these patients are usually overweight or obese. The diagnosis of youth‐onset type 2 diabetes was confirmed in the present patient, and other types of diabetes mellitus were excluded.

The proband underwent insulin and metformin treatment, performed also at high doses, not allowing however to limit glycosuria and ketonuria even when blood glucose levels almost reached the normal range. Then, a possible concurrent FRG diagnosis was supposed.

FRG is a rare renal tubular disorder in which patients present isolated persistent glycosuria without abnormal glucose metabolism and other proximal tubular disorder. Previous studies confirmed that *SLC5A2* gene mutations are responsible for FRG. Most of patients with heterozygous mutations have slight glycosuria (<10 g/d) or no glycosuria, whereas other individuals with homozygous or compound heterozygous mutations usually have serious glycosuria (>10 g/d).^7^ Qian Ren research showed that the urine glucose excretion was significantly higher in patients with homozygous mutations than those with compound heterozygous ones.^8^ To date, 115 variants in the *SLC5A2* gene were identified. The most frequent ones include missense, frameshift, and splicing mutations.^9^ They appear to affect transport activity through decreasing intrinsic transporter activity, reducing protein insertion into cell membrane, inhibiting protein synthesis, and accelerating protein degradation. FRG is a benign condition, which does not need any specific treatment, because it causes no apparent symptoms or severe outcomes, such as hypoglycemia, polyuria, or dehydration.

In this family, a heterozygous variation was detected in the *SLC5A2* gene (NM_003041.4:c.1129 + 2 T > C) in the proband and her father. The novel mutation occurred at the exon‐intron boundaries. Theoretically, the mutation can affect the normal cleavage of mRNA. To date, this variation has not been reported in literature or in large‐scale population frequency databases. According to the clinical manifestations presented in the proband, we considered the *SLC5A2* mutation detected as likely pathogenic, based on the variation classification guidelines of ACMG. SGLT2 plays a key role in sugar binding and translocation in proximal convoluted tubule. Its mutation can lead to SGLT2 impaired function, decreasing the reabsorption of glucose from the filtered urine and reducing blood glucose together with sodium depletion, and being thus responsible for glycosuria and osmotic diuresis. As a result, this youth‐onset type 2 diabetic patient also had continuous glycosuria. Theoretically, we hypothesized the hyperglycemia is more easier to be controlled in type 2 diabetes patients coexisting with FRG. However, this phenomenon was not observed in this patient, because her blood glucose levels were not well controlled with a higher dose of insulin combined with metformin. It has been hypothesized that, because lipolysis within the adipose tissues and the free fatty production acids will increase, subsequently an uninhibited transport of fatty acids into hepatic mitochondria and ketone bodies production through ß‐oxidation may occur.^10^ Actually, SGLT2 inhibition can directly act on pancreatic α‐cells to increase secretion of glucagon, leading to the propensity of ketone production. *SLC5A2* gene mutations and then SGLT2 dysfunctions might be responsible for glucagon hypersecretion, thus causing continuous glycosuria and ketonuria even when blood glucose is near the normal range.

The proband and her father had different phenotypes even though they had the same mutation. Such variants are referred to as incomplete penetrance, leading to a wide range of diverse phenotypes, from asymptomatic cases to severe disease among related individuals. Both incomplete penetrance and variable expressivity are thought to be caused by many factors, including common variants, variants in regulatory regions, epigenetics, environmental factors, and lifestyle.^11^, ^12^ The inheritance of FRG is usually confirmed as codominant with incomplete penetrance, because not all patients with similar or identical mutations have the same degree of excessive glucose excretion.^13^ Our finding was consistent with the literature reports.

Additionally, the patient suffered from recurrent UTI. Diabetic women have an increased incidence of recurrent UTI compared with nondiabetic patients, as excessive glucose in the urine can enhance bacterial growth. It may be worse in our diabetic female patient also affected with FRG, because glycosuria and consequent predisposition to growth of commensal microorganisms were more serious.

The etiologies of diabetes mellitus are different, and diabetic patients usually present with many characteristics. Thus, the differential diagnosis is usually difficult, especially when diabetic patients concurrently suffer from other rare diseases, which may interfere with glucose homeostasis as observed in the present patient. Clinicians should enhance their ability to distinguish such complex phenotypes and perform early and careful clinical evaluation in these patients.^14^, ^15^ Genetic investigations, including next generation sequencing, are extremely useful in identifying specific gene mutations in the patients, or in their family members, as well as to ensure the most appropriate therapy and follow‐up.^16^, ^17^, ^18^, ^19^

## AUTHOR CONTRIBUTIONS

Yuqing Qu and Xianling Wang conceived of and designed the work. Limei Hao acquired the data. Yuqing Qu analyzed the data and drafted the manuscript. Xianling Wang critically revised the manuscript for important intellectual content. All authors were responsible for the interpretation of the data and revised and gave final approval of the manuscript. Xianling Wang is the guarantor of the work and, as such, had full access to all the data and takes responsibility for the integrity of the data and the accuracy of the data analysis.

## FUNDING INFORMATION

This study did not receive any specific grant from funding agencies in the public, commercial, or not‐for‐profit sectors.
